# Application of Bacteriophages for Human Health: An Old Approach against Contemporary “Bad Bugs”

**DOI:** 10.3390/microorganisms10030485

**Published:** 2022-02-22

**Authors:** Lucia Henrici De Angelis, Greta Ponsecchi, Maurizio Fraziano, Marco Maria D’Andrea

**Affiliations:** 1 Department of Medical Biotechnologies, University of Siena, 53100 Sienna, Italy; lucia.henrici@gmail.com; 2 PhD Program in Evolutionary Biology and Ecology, Department of Biology, University of Rome “Tor Vergata”, 00133 Rome, Italy; gretaponsecchi@gmail.com; 3 Department of Biology, University of Rome “Tor Vergata”, 00133 Rome, Italy; fraziano@bio.uniroma2.it

The breadth of the antimicrobial resistance (AMR) problem exposes humankind to serious threats, which could lead, in the near future, to a worrisome raising of mortality and morbidity rates due to infections by “bad bugs”. Indeed, a previous report on AMR commissioned by the UK government suggested that this phenomenon was responsible for a minimum of 700,000 deaths in 2014 and that this estimate could rise to 10 million per year by 2050 if no reasonable efforts are carried out [1]. In line with this concern, a recent study evaluating the burden due to AMR for 2019, although considering a wider number of pathogen–drug combinations than those evaluated by O’Neill [1], provides higher estimates and clearly underscores once again that AMR is a leading cause of death at the global scale [2]. On this basis, it is apparent that novel strategies aimed to prevent and treat diseases due to pathogenic bacteria must be a primary aim of both basic and applied research. A possible option to tackle bacterial infections, which has recently gained renewed interest, is the use of phages or their components to prevent or treat human infections. This idea, which was already explored by d’Hérelle soon after the discovery of bacterial viruses, is currently being widely investigated by many scientists given the several advantages it exhibits [3]. Indeed, bacteriophages may be specific “killers” of a single bacterial species or even of single clonal lineages, thus representing a potentially powerful tool for targeted interventions able to leave the beneficial human microbiota largely undisturbed. Moreover, phages are the most abundant biological entities on our planet, and the isolation of candidates with features suitable for clinical uses and their characterization is relatively an easy, fast and cheap process. In addition, some peculiarities such as (i) the ability to replicate themselves where the targeted bacteria are present; (ii) their immunogenicity in humans, which is usually not clinically relevant and scarcely affects their effectiveness in terms of bacterial killing, and (iii) the ability to often exploit bacterial pathogenicity factors as receptors make phages very interesting and promising weapons in the fight against antibiotic resistant bacteria and, in general, against “bad bugs”.

This Special Issue collects several original works contributing in this field and also some review papers summarizing the state of the art on (i) the use of phages in different scopes, including the decontamination of inanimate surfaces [4]; (ii) the role of animal models to determine the safety and effectiveness of phage therapy, in particular considering treatments against some major multidrug resistant (MDR) bacteria [5]; (iii) the occurrence and role of phages in the human urinary tract [6]; and (iv) several aspects of phage-based inhibition of *Listeria monocytogenes* in food matrices [7]. In particular, two excellent contributions [4,7] highlighted that the use of commercial bacteriophage products as biocontrol agents has already been approved by some regulatory authorities for direct use in agriculture to prevent specific plant diseases or on some foods. The use of these formulations, which was granted a GRAS status (generally recognized as safe) by several government authorities, significantly spurs the direct use of phages as therapeutic agents in humans, which is currently rather limited to specific sporadic cases in most countries.

At the same time, this collection reports many descriptions of newly characterized phages, which could be good candidates for the treatment of human infections due to *Enterococcus faecalis* [8,9], *Klebsiella pneumoniae* [10], *Escherichia coli* [11] and *Pseudomonas aeruginosa* [12] or to combat phytopathogens [13,14] or bacteria responsible for foodborne diseases [15,16,17]. It is relevant to underscore that, besides the use of standard techniques for general characterization of phages, some contributions evaluated their effective activity on bacterial biofilm [8,11,12] or on animal infection models [9,10], while others took original experimental approaches tailored to the future intended usage of the investigated phages [8,13,16]. Altogether, these models constitute major steps forward for the applicability of phages for therapeutic purposes or, more generally, as biocontrol agents.

Other scientific contributions within this issue present original uses of phages, such as their employment as a fast, highly sensitive and very specific tool for the fluorometric detection of *E. coli* contaminants in ground beef [18] or as an agent to control colonization and infection by *Helicobacter pylori* while minimizing the damage of the host’s proinflammatory response to infection by the synergistic action of lactoferrin [19]. Additionally, the work by Núñez-Sánchez et al. [20] presents an intestinal epithelium model endowed with a mucus layer to assess the therapeutic value of a newly isolated bacteriophage and to characterize the interaction between the phage and opportunistic pathogens such as *E. faecalis*. This model has an interesting advantage that allows for the evaluation of the interaction of phages with target bacteria in a complex environment, mimicking that of the human host, thus also allowing the evaluation of the effect of treatment on eukaryotic cells of the gut. In their article, Penttinen et al. [21] discussed the use of phages targeting pili encoded on some conjugative resistance plasmids. Such an approach would have the peculiar ability to cross the species or clonal lineage boundaries usually observed with the use of phages, providing a way to kill plasmid-carrying bacteria regardless of their specific taxonomical position. Martin et al. [22] characterized the antibiotic and phage susceptibility of longitudinal isolates of *P. aeruginosa* from cystic fibrosis patients to a commercial phage cocktail. Their work underscores that, similar to what happens with antibiotics, there is a need to periodically assess the action of phages on target bacteria when used as therapeutic tools, possibly using several colonies from the same sample. Lastly, Henrici De Angelis et al. [23] characterized the receptor used by a lytic phage specific to a major clonal lineage of MDR *K. pneumoniae*, showing that phage-resistant mutants not expressing the receptor exhibited a lower pathogenicity level in a *Galleria mellonella* infection model. Their findings, which are in line with those of previous literature, suggest that the emergence of phage-resistant mutants could lead to reduced virulence in the host; therefore, this event could be beneficial for the host. Collectively, these works expand the number of candidate bacteriophages that can be used against pathogenic bacteria in different fields of application, ranging from therapy for human infections to agricultural and food applications.

In conclusion, it could be envisioned that phage therapy will constitute a relevant weapon, in the near future, for the battle against human bacterial pathogens, especially considering the fast loss of effectiveness of the current antimicrobial armamentarium we are facing. To this aim, besides efforts of basic research, it is imperative to increase the currently low number of validated and rigorous clinical trials to extend the clinical use of phages, which is currently limited to selected complex cases. The characterization of novel bacteriophages, their use in combination with other antimicrobial agents or immunostimulators able to spur the host immune system [24], the definition of thoroughly characterized phage models to be engineered for different purposes and the study of specific phage components to be used in place of entire phages could be other promising and innovative approaches to translating the employment of phages in clinical uses. Such research needs timely, coordinated and multidisciplinary political and social efforts in order to be stimulated and funded by specific programs undertaken at the international level.

## Data Availability

Not applicable.
